# Inter‐assemblage facilitation: the functional diversity of cavity‐producing beetles drives the size diversity of cavity‐nesting bees

**DOI:** 10.1002/ece3.1871

**Published:** 2016-01-08

**Authors:** Markus A. K. Sydenham, Lise D. Häusler, Stein R. Moe, Katrine Eldegard

**Affiliations:** ^1^ Department of Ecology and Natural Resource ManagementNorwegian University of Life SciencesP. O. B. 5003ÅsNO‐1432Norway

**Keywords:** Cavity nesting bees, community assembly, community ecology, facilitation, functional diversity, functional traits, pollinators, wild bees, wood boring beetles

## Abstract

Inter‐specific interactions are important drivers and maintainers of biodiversity. Compared to trophic and competitive interactions, the role of non‐trophic facilitation among species has received less attention. Cavity‐nesting bees nest in old beetle borings in dead wood, with restricted diameters corresponding to the body size of the bee species. The aim of this study was to test the hypothesis that the functional diversity of cavity‐producing wood boring beetles ‐ in terms of cavity diameters ‐ drives the size diversity of cavity‐nesting bees. The invertebrate communities were sampled in 30 sites, located in forested landscapes along an elevational gradient. We regressed the species richness and abundance of cavity nesting bees against the species richness and abundance of wood boring beetles, non‐wood boring beetles and elevation. The proportion of cavity nesting bees in bee species assemblage was regressed against the species richness and abundance of wood boring beetles. We also tested the relationships between the size diversity of cavity nesting bees and wood boring beetles. The species richness and abundance of cavity nesting bees increased with the species richness and abundance of wood boring beetles. No such relationship was found for non‐wood boring beetles. The abundance of wood boring beetles was also related to an increased proportion of cavity nesting bee individuals. Moreover, the size diversity of cavity‐nesting bees increased with the functional diversity of wood boring beetles. Specifically, the mean and dispersion of bee body sizes increased with the functional dispersion of large wood boring beetles. The positive relationships between cavity producing bees and cavity nesting bees suggest that non‐trophic facilitative interactions between species assemblages play important roles in organizing bee species assemblages. Considering a community‐wide approach may therefore be required if we are to successfully understand and conserve wild bee species assemblages in forested landscapes.

## Introduction

In community ecology we tend to study the processes related to the diversity within a single species assemblage (Fauth et al. 1996; Lawton 1999) such as competition and trophic interactions (Potts et al. 2003) or the impact of environmental filters on the functional and phylogenetic diversity of bee species assemblages (Hoiss et al. 2012). A species assemblage is here defined following Fauth et al. (1996) as those species found within a community that belong to the same taxa. However, the diversity within species assemblages may also depend on non‐trophic facilitative interactions with other species assemblages in the same community through processes of ecosystem engineering, whereby some species alter the environment in ways that opens niches for other species to occupy (Jones et al. 1994; Lawton 1994; Bruno et al. 2003). Improving our understanding of the influence of such interactions may have considerable bearing on the successful management of habitats that host species assemblages of conservation priority.

Wild bees have received increased attention over the last two decades due to declines in pollinator diversity worldwide (Potts et al. 2010) and their expected impact on seed production in domesticated (Klein et al. 2007) and wild plants (Fründ et al. 2013), where an estimated 87.5% of wild angiosperms are pollinated by animals (Ollerton et al. 2011). Indeed, the influences of many important drivers on the diversity of wild bees in anthropogenic landscapes are well documented (Winfree et al. 2011). Bees rely on forage resources, nest sites and nest building materials, each of which are sometimes found in separate habitats (Westrich 1996). The diversity of both foraging (Potts et al. 2003; Müller et al. 2006) and nesting resources (Potts et al. 2005; McFrederick and LeBuhn 2006a,b; Murray et al. 2012) contribute to structuring wild bee species assemblages at the local scale. At the landscape scale a shortage of habitat types providing these resources partly explains the variation in diversity between bee species assemblages (Steffan‐Dewenter et al. 2002; Hopfenmüller et al. 2014). In addition to resource‐related habitat conditions, large‐scale environmental filters such as differing climatic and nesting conditions along elevational gradients also play an important role in structuring wild bee species assemblages (Hoiss et al. 2012; Sydenham et al. 2015).

In the course of the past decade, there has been an increased focus on the role functional traits play in organizing species assemblages (Weiher et al. 2011). Functional diversity indices may reveal mechanistic links between biodiversity and ecological processes (Petchey and Gaston 2006; Laliberté and Legendre 2010; Ricotta and Moretti 2011) which may not be found if one relies solely on indices based on the species richness and abundances of individuals (Cadotte et al. 2011). Indeed, the consequences of land‐use change for wild bee species assemblages depend on the functional traits of bee species such as nesting habits (Williams et al. 2010; Hopfenmüller et al. 2014). One functional trait‐group is the cavity‐nesting bees, here defined as solitary bees that nest in pithy stems as well as abandoned beetle burrows in dead wood. In meadows, the diversity of cavity‐nesting bee species assemblages is higher in sites containing old fruit trees compared to sites lacking of old trees (Tscharntke et al. 1998), suggesting that nesting substrates may be a limiting factor for these bees (Steffan‐Dewenter and Schiele 2008).

During the past century, silviculture has reduced the amount of dead wood in forests by as much as 90–98% in some areas (Siitonen 2001), leading to the regional extinctions of several species of wood boring beetles (Grove 2002). In Norway, 40% of the red listed beetle species depend on forest habitats and dead wood (Kålås et al. 2010). Wood boring beetles play an important function in forested landscapes by excavating cavities in dead wood, which ‐ once abandoned ‐ go on to be occupied by other species of cavity‐nesting insects such as bees (Ehnström and Axelsson 2002; Stokland et al. 2012). Although many species of Hylaeine and Megachiline bees (hereafter referred to as cavity‐nesting bees) nest in abandoned beetle nests in dead wood (Westrich 1989), and that some bee species are directly associated with forests (Winfree et al. 2007), the influence of the diversity of wood boring beetles on the diversity of cavity‐nesting bees has received little attention (but see Westerfelt et al. 2015). For instance, as cavity‐nesting bee species vary in body‐size and prefer different diameters of potential nest‐sites (Gathmann et al. 1994; Tscharntke et al. 1998), they are likely to nest in holes produced by different species of beetles. Understanding the properties of such relationships may be of high importance for the conservation of bees if historical reductions of dead wood have had cascading effects on cavity‐nesting bees by initially reducing the diversity of wood boring beetles. Moreover, compared to artificial nests, only a small proportion of natural beetle borings are occupied by bees and other Aculeates (Westerfelt et al. 2015), suggesting that the quality of the nesting substrate is of high importance when bees evaluate the suitability of a nesting site. It is therefore possible that the functional diversity of freshly emerged wood boring beetles provides an informative surrogate for the availability and diversity of recently excavated, i.e. high quality, cavities in an area. We formulated three hypotheses allowing us to infer if the diversity of wood boring beetles is an important determinant of cavity nesting bee diversity.

*Hypothesis 1:* The species richness and abundance of cavity nesting bees show a significant increase with the species richness and abundance of wood boring beetles. A similar relationship is not expected for the species richness and abundance of non‐wood boring beetles or wood boring beetles that produce cavities that are too small for bees to occupy. Additionally, the influence of the species richness and abundance of cavity producing beetles is not driven by a co‐variation with other environmental filters, such as elevation or the area of similar habitat (i.e. width of the power line clearing).
*Hypothesis 2*: The positive associations between cavity nesting bees and cavity producing beetles are driven by nest‐site facilitation and not by shared positive responses to underlying foraging resources, such as floral diversity, which should also favour non‐cavity nesting bees. An increased cavity‐producing beetle species richness and abundance should therefore lead to an increased proportion of cavity nesting bees in local bee species assemblages. This relationship should be significant even when the nesting conditions for ground nesting bees are accounted for (i.e. the degree of shading due to regrowth).
*Hypothesis 3*: The occupation of beetle borings by cavity nesting bees depends on the diameter of the boring, and the size of the bee. An increased species richness, abundance and functional diversity (in terms of boring diameters) of wood boring beetles in the forested landscape should provide a higher diversity of nesting spaces and lead to a high size diversity (in terms of body sizes) of cavity‐nesting bees.

## Materials and Methods

### Study system and sampling

The study was conducted in 30 power line clearings (mean width = 42 m, SD = 18 m) along an elevational gradient (36–568 m a.s.l.) in a landscape dominated by boreal forests with varying proportions of the main tree species: Norway spruce *Picea abies*, Scots pine *Pinus sylvestris* and birch *Betula* spp. (Fig. S1, Supporting information). Power line clearings are typically situated in areas of low to intermediate productivity and cleared every 5–10 years to prevent trees from encroaching on the aerial lines. Establishing and maintaining power line clearings creates “through corridors” of earlier successional vegetation and long, often sharply defined, permanent edges on either side of the clearing (Eldegard et al. 2015). Edge creation and selective felling of tall trees leads to increased tree mortality and a greater abundance of snags and logs at edges (Harper et al. 2005, pers. obs.). It is therefore likely that dead wood is less of a limiting factor for wood boring beetles in these habitats than in the intensively managed forests although dead wood is also found in these habitats (e.g. in the form of stumps in recently cleared forests). The system thereby creates a good model‐system for evaluating the role of wood boring beetles as facilitators for cavity‐nesting bees.

Sampling was conducted at 30 different sites in, respectively, 2009 (10 sites), 2010 (10) and 2013 (10). Inter‐site distances (mean = 83 km, min = 9 km, max = 187 km) were greater than the foraging range of the bees (Gathmann and Tscharntke 2002; Greenleaf et al. 2007) ensuring independency among bee species assemblages. Beetles and bees were sampled using flight‐interception traps, which allowed for a standardized sampling at several sites covering a large geographic area. Flight interception traps are commonly used to sample beetles (Økland 1996) and have been especially recommended for the collection of wood‐nesting bees (Rubne et al. 2015). Each trap consisted of two Plexiglas screens (370 × 210 mm) assembled to form a cross attached to a white funnel with a collecting bottle attached to it. The bottle was filled with a 50:50 mixture of green propylene glycol and water plus a few drops of detergent to break the surface tension. Four traps were deployed along the centre of the power line clearings in each of the 10 sites sampled in 2009 and 2010. Since some traps were destroyed during the sampling periods in 2009 and 2010 the number of traps per site was increased to five in 2013. The traps were installed following snow‐melt (April/May) and removed in the early autumn (August/September). The traps were emptied four times during the trapping season and the collected material stored in 80% ethanol before pinning and identification.

We placed four 4 × 5 m plots along the centre of the power line clearing, following the direction of the corridor. The distance between the two nearest plots was 50 m. Within each of the four plots, we recorded the total number of tree species, the tree height and the crown of all species taller than one metre. In addition to the measures of tree numbers and sizes, we also recorded the basal area (relascope sum) and site productivity (see Eldegard et al. (2015)). For each site, we calculated the following variables: The total number of coniferous, Norway spruce, Scots pine and deciduous trees as well as the total number of trees. We also calculated the average height and crown width of trees within the sites as well as the maximum tree height, crown width and productivity recorded in any one of the four plots. Together these variables described the amount of regrowth and productivity and hence shading within the site. To deal with collinearity among these variables, we condensed them into two principal components. The variables were scaled to zero mean and unit deviance. Thereafter, the scaled variables were run through a Principal Components Analysis (PCA) using the “vegan” library in R (Oksanen et al. 2013) whereby we extracted the two‐first axes. The Eigenvalues and proportion variation explained were 5.54 and 50.4% and 2.45 and 22.5% for PCA axis one and two, respectively. PCA axis one was positively related to all the variables and thereby indicated a gradient in regrowth (i.e. shading). PCA axis two was positively related to the total number of trees, number of deciduous trees, number of spruce trees as well as the site productivity and weakly related to the maximum crown width, and negatively related to the number of coniferous trees, pine trees, average and maximum tree height, basal area and crown cover. It thereby separated sites according to productivity and along a successional gradient being positively related to regrowth of trees in the clearings (Table S1, Supporting information). We extracted the site scores on the first two PCA axes and used these as variables to explain the effect of shading.

### Statistical analyses

In order to compensate for traps lost during the sampling periods, a subset of traps were randomly selected and removed from each sampling period so that the sampling intensity within sites was equal across all sites and years. Three sites had lost more than one trap during a sampling period and were removed from the final dataset. The final dataset consisted of 27 sites each sampled with three traps during the first sampling period, four traps during the second and third and three traps during the final sampling period. All specimens collected in a site were pooled and sites were used as sampling units in the analyses. Cleptoparasitic bees were excluded from the analyses as they only indirectly depend on the nesting and foraging resources sought by their hosts.

The beetles were categorized into four groups. The first group consisted of “all beetles” sampled in the study. The second group consisted of all non‐wood boring beetles. The third group consisted of small wood boring beetles that excavate cavities in dead wood with a diameter smaller than 3 mm or in the roots of plants. The fourth group consisted of large wood boring beetles that are known to excavate cavities with a diameter >3 mm in wood (Ehnström and Axelsson 2002). The distinction between the small and the large wood boring beetles was made because only beetles making holes, above ground, with diameters ≥3 mm are producers of possible nesting holes for the cavity nesting bees in our region (Budrien≐ et al. 2004; Westerfelt et al. 2015). All large wood boring beetle species were assigned to a diameter class equal to the diameter of the exit holes produced by the emerging adults (Ehnström and Axelsson 2002). Cavity nesting bees were grouped according to their thorax width (Table S2, Supporting information), since body size determines the minimum diameter of cavities in which they nest.

Three metrics that together account for the size diversity within bee and the functional diversity within beetle species assemblages were used in order to assess whether an increased functional diversity of wood boring beetles leads to a high size diversity of cavity‐nesting bees. The functionally singular species richness (FSSR) is the number of unique size types found within the assemblage. As such it is the functional equivalent of nomenclatural species richness. The community weighted mean trait value (CWM) is a measure of the dominant trait value within a species assemblage. The functional dispersion (FDis) is a measure of the variation in trait values within a species assemblage (Laliberté and Legendre 2010). When the FDis is based on a single, numerical trait, it equals the mean absolute Euclidean distance of trait values found within the species assemblage to the CWM. These metrics were chosen since changes in both the dispersion of trait values and the CWM have been shown to be informative metrics for studies on functional bee ecology (Ricotta and Moretti 2011). Since an increase in the functional diversity of wood boring beetles should lead to an increased diversity and accessibility of nesting niches for bees, it should be expected that an increase in the FSSR, FDis and CWM of wood boring beetles would lead to an increase in FSSR, FDis and CWM of cavity nesting bees. The R (R development core team 2014) library “FD” (Laliberté and Legendre 2010) was used to calculate the size class richness (FSSR_bees_), the community‐weighted mean size (CWM_bees_) and the dispersion of size classes (FDis_bees_) for the cavity nesting bees. For the large wood boring beetles the CWM and the FDis were weighted according to the abundance of each species. The measures for bees were not abundance‐weighted as doing so might decrease the influence of the relatively large Megachilids. Although species within this family were relatively rare, compared to the most abundant *Hylaeus* species, their presence within species assemblages provide important information about the niche‐space occupied by bees in the species assemblage. However, the non‐abundance weighted measures of both the FDis_bees_ and the CWM_bees_ were highly correlated with their abundance weighted counterparts (rho = 0.96, *P* < 0.001 and rho = 0.97, *P* < 0.001, respectively) suggesting only a small influence of abundance weighting the indices.

Due to the presence of multicolinearity among the explanatory variables (Table S3, Supporting information), the influence on response variables of each of the explanatory variables were analysed individually and the strengths of significant relationships assessed based on the Nagelkerke *R* ^2^, standardized effect sizes and *P*‐values. This approach allowed an evaluation of the direct influence of each explanatory variable separately, in contrast to solely evaluating its influence based on the marginal effect as would be the case were it tested simultaneous with other variables. Variables with *P*‐values ≤0.10 were then included in a full model which was subjected to a step‐wise backward elimination based on likelihood ratio tests (LRTs) by dropping variables one at a time until all the remaining variables were significant (*P* ≤ 0.05).

### The species richness and abundance of cavity nesting bees increase with the species richness and abundance of large wood boring beetles (Hypothesis 1)

The association between cavity nesting bees and large wood boring beetles was compared to the association with small wood boring beetles, non‐wood boring beetles and elevation. The individual influences of the explanatory variables on cavity nesting bee species richness were tested using generalized linear models (GLMs) with Poisson distributed errors. For the abundance of cavity nesting bees over‐dispersion was accounted for by using negative binomial regressions in the “MASS” library (Venables and Ripley 2002) in R. Sampling year was included as a categorical variable with three levels (2009, 2010 and 2013) to account for potential among‐year differences in cavity nesting bee species richness and abundance due to inter‐annual climatic variations. Model fit was assessed from Nagelkerke *R* ^2^ values. In addition to comparing the influence of the explanatory variables based on their standardized effect sizes (*z*‐values), Nagelkerke *R* ^2^ values and *P*‐values a backward elimination of explanatory variables was conducted to allow a formal comparison of variables based on their marginal effects. Candidate models, consisting of all explanatory variables with *P* < 0.10, were subjected to backward elimination of variables. The relative importance of each variable was tested using likelihood ratio tests. One by one the variables with the lowest *χ* ^2^ score and highest *P*‐value were removed from the model until all variables in the final model were significant (*P* < 0.05).

We also tested if the species richness and abundance of cavity nesting bees and large wood boring beetles showed significant relationships with the width of the power line clearing. This was done to test the assumption that a positive association among cavity nesting bees and wood boring beetles was not driven by similar species‐area relationships. We used the width of the power line clearing as an explanatory variable indicating the area of similar habitat conditions and the species richness and abundance of either cavity nesting bees or large wood boring beetles as response variables. The analyses with species richness response variables were run using GLMs, assuming Poisson distributed errors whereas the analyses with abundances as response variables were run using negative binomial regressions.

### The proportion of cavity nesting bees in bee species assemblages increases with the species richness and abundance of large wood boring beetles independent of vegetation shading the ground (Hypothesis 2)

For each site, the proportion of cavity nesting bee species and individuals were calculated relative to the total number of non‐cavity nesting bee species and individuals. Since the cavity nesting bees in our study sites mainly forage on forbs rather than dwarf shrubs (Ericaceae), we also calculated the proportion of cavity nesting bee species and individuals for each site when the dwarf shrub (Ericaceae) specialists *Andrena fuscipes, A. lapponica* and *Colletes succinctus* were removed from the data, resulting in a total of four response variables.

The relationships between the proportion of cavity nesting bee species and individuals and the species richness and abundance of large wood boring beetles and sampling year were assessed using binomial generalized linear models (GLMs). We also included the two PCA axes related to shading and site productivity to account for potentially contrasting responses of cavity nesting and ground nesting bees to regrowth, which might prevent ground nesting bees from nesting in the site. The significance of each explanatory variable was tested separately using likelihood ratio tests. All explanatory variables with *P*‐values ≤ 0.10 were included in a full model and subjected to a manual step‐wise backward elimination until all variables were statistically significant (*P* ≤ 0.05).

### The functional diversity within beetle species assemblages drives the size diversity within bee species assemblages (Hypothesis 3)

The relationship between the size diversity of cavity nesting bees and the species richness, abundance and functional diversity of large wood boring beetles was compared to the relationship with the species richness, abundance of non‐wood boring beetles, small wood boring beetles and elevation. Response variables were the number of unique bee size classes (FSSR_bees_), the community‐weighted mean (CWM_bees_) body size and the variation in body sizes (FDis_bees_) in each site. Explanatory variables were the species richness, abundance, CWM and FDis of large wood boring beetles, the species richness and abundance of non‐wood boring beetles and small wood boring beetles and elevation. The sampling year was included as a categorical variable with three levels (2009, 2010 and 2013).

Analyses with the FSSR_bees_ as response variables were conducted using GLMs with Poisson distributed errors. The analyses with the CWM_bees_ and the FDis_bees_ as responses were conducted using quasipoisson GLMs. The models with CWM of large wood boring beetles (L WB B) as explanatory variables were fitted using the second order polynomial to account for the hump‐backed relationship with the response variable, which was detected in the exploratory analyses of the data. Since the size diversity could not be calculated for sites where no bees were sampled, two sites were omitted from these analyses. The influence of the explanatory variables was assessed based on to their Nagelkerke *R* ^2^, *z*‐values and their *P*‐values (*α *= 0.05).

## Results

A total of 621 species and 14,609 individuals of beetles and 47 species and 354 individuals of solitary and primitively eusocial bees were sampled. Sixty‐five species and 1974 individuals of the beetles were woodborers. Of the wood boring beetles, 18 species and 791 individuals produced cavities in wood with a diameter ≥3 mm in which bees may nest (Table S2, Supporting information). Of the bees 15 species and 147 individuals were cavity nesters, and 9 species and 20 individuals were clepto‐parasites (Table S4, Supporting information).

### The species richness and abundance of cavity nesting bees increase with the species richness and abundance of large wood boring beetles (Hypothesis 1)

**Figure 1:**
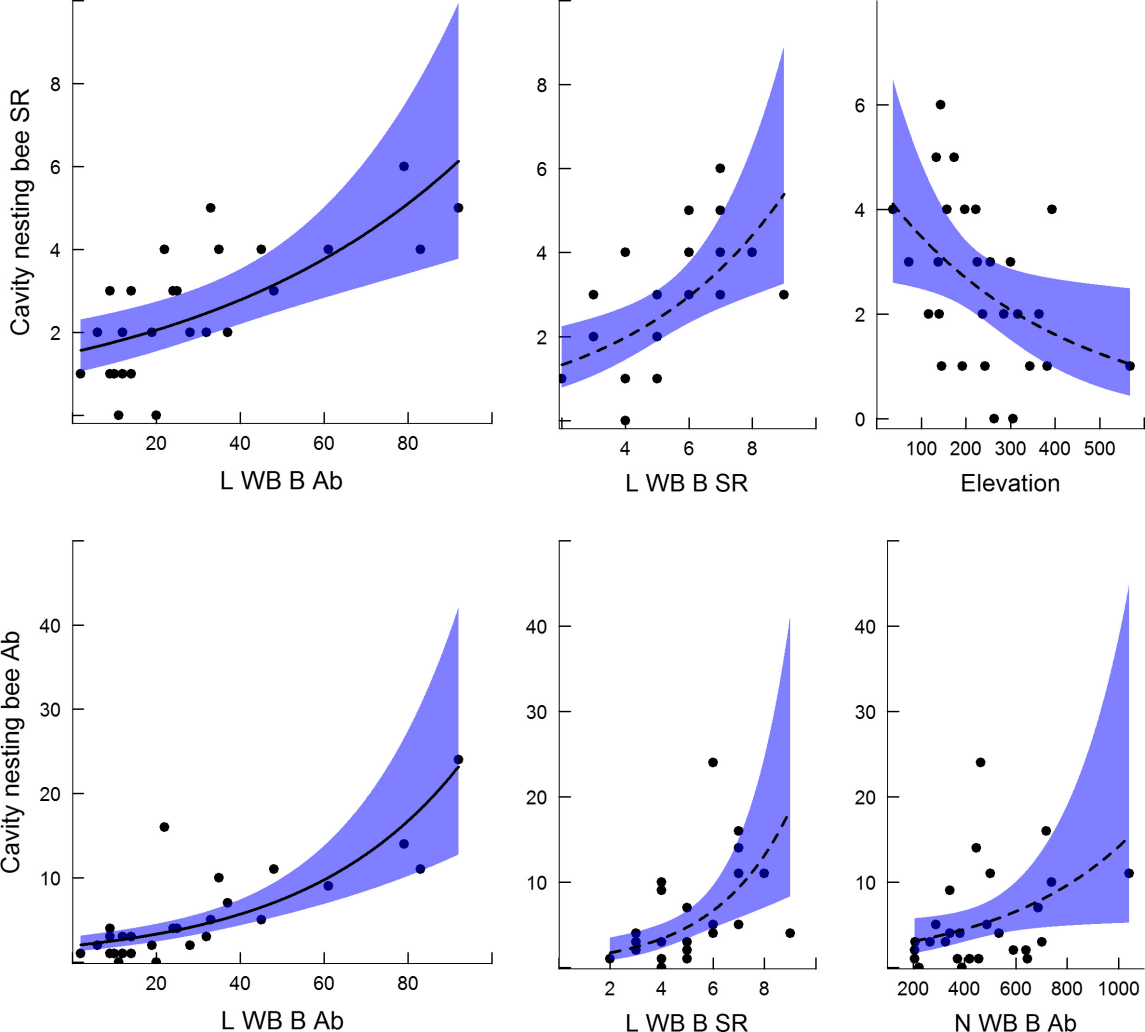
The species richness (SR) of cavity nesting bees (top panel) was influenced by the abundance (Ab) and species richness (SR) of large wood boring beetles (L WB B) which excavate cavities with diameters ≥3 mm and elevation (m a.s.l.). Similarly, the abundance of cavity nesting bees (lower panel) was related to the L WB B Ab, L WB B SR but also the abundance of non‐wood boring beetles (N WB B Ab). Enlarged plots with solid regression lines show estimated values for the explanatory variables remaining after backward elimination (see text and Table 1 for test statistics).

The species richness of cavity nesting bees increased with the species richness (Fig. 1, df = 1, *χ* ^2^ = 8.85, *P* = 0.003) and abundance (Fig. 1, df = 1, *χ* ^2^ = 12.01, *P* = 0.001) of large wood boring beetles and decreased with elevation (Fig. 1, df = 1, *χ* ^2^ = 5.09, *P* = 0.024). The abundance of large wood boring beetles was the most important variable explaining cavity nesting bee species richness and was the only explanatory variable left in the final model, following backward elimination (Table 1). In contrast the species richness of cavity nesting bees was not influenced by the species richness (df = 1, *χ* ^2^ = 2.31, *P* = 0.129) or abundance (df = 1, *χ* ^2^ = 0.73, *P* = 0.394) of small wood boring beetles or by the species richness (df = 1, *χ* ^2^ = 0.52, *P* = 0.470) or abundance (df = 1, *χ* ^2^ = 2.39, *P* = 0.123) of non‐wood boring beetles. Nor were there any significant difference in cavity nesting bee species richness among years (df = 2, *χ* ^2^ = 3.99, *P* = 0.136).

**Table 1:** Final models for cavity nesting bee species richness and abundance following backward elimination of the explanatory variables with a *P*‐value <0.10 (Table S5, Supporting information). The model for bee species richness was fitted using GLMs with Poisson distributed errors, while the influence of explanatory variables on bee abundance was modelled using negative binomial regressions to account for overdispersion. Large wood boring beetles excavate cavities with diameters ≥3 mm suitable for cavity nesting bees. Nagelkerke *R* ^2^ values are shown

	df	*β*	SE	*z* value	*R* ^2^	*P*‐value
Cavity nesting bee species richness						
Intercept	25	0.417	0.206	2.03		0.043
Large wood boring beetles abundance		0.015	0.004	3.65	0.54	<0.001
Cavity nesting bee abundance						
Intercept	25	0.658	0.216	3.04		0.002
Large wood boring beetles abundance		0.027	0.005	5.73	0.80	<0.001

The abundance of cavity nesting bees increased with the species richness (Fig. 1, df = 1, *χ* ^2^ = 11.97, *P* = 0.001) and abundance (Fig. 1, df = 1, *χ* ^2^ = 35.17, *P* < 0.001) of large wood boring beetles, and the abundance of non‐wood boring beetles (Fig. 1, df = 1, *χ* ^2^ = 4.33, *P* = 0.037) when each explanatory variable was tested separately. However, the abundance of large wood boring beetles was the most influential explanatory variable and the only variable remaining in the negative binomial regression model following backward elimination of variables (Table 1). We did not find a significant change in cavity nesting bee abundance with elevation (df = 1, *χ* ^2^ = 3.10, *P* = 0.079) or among years (df = 2, *χ* ^2^ = 4.91, *P* = 0.086), but the *P*‐values were low. We found no effect on cavity nesting bee abundance of small wood boring beetle species richness (df = 1, *χ* ^2^ = 1.04, *P* = 0.307) or abundance (df = 1, *χ* ^2^ = 1.07, *P* = 0.302) or non‐wood boring beetle species richness (df = 1, *χ* ^2^ = 0.16, *P* = 0.686). See Table S5 (Supporting information) for test statistics and parameter estimates for the individual explanatory variables and full models.

The significant relationship between cavity nesting bee species richness and abundance and the species richness and abundance of large wood boring beetles was not due to co‐variation with habitat area: we found no relationship between the species richness of cavity nesting bees (df = 1, *χ* ^2^ = 0.09, *P* = 0.762) or large wood boring beetles (df = 1, *χ* ^2^ = 1.04, *P* = 0.307) and the width of the power line clearing. Nor did we find any significant relationship between the abundance of cavity nesting bees (df = 1, *χ* ^2^ = 0.12, *P* = 0.728) or large wood boring beetles (df = 1, *χ* ^2^ = 0.27, *P* = 0.605) and the width of the power line clearing.

### The proportion of cavity nesting bees in bee species assemblages increases with the species richness and abundance of large wood boring beetles independent of vegetation shading the ground (Hypothesis 2)

The proportion of cavity nesting bee species in local bee species assemblages did not change significantly according to the species richness (df = 1, *χ* ^2^ = 1.19, *P* = 0.28) or abundance (df = 1, *χ* ^2^ = 0.02, *P* = 0.89) of large wood boring beetles. Nor did it vary among sampling years (df = 2, *χ* ^2^ = 0.45, *P* = 0.80) or with vegetation shading the ground (PCA axis one; df = 1, *χ* ^2^ = 1.95, *P* = 0.16, PCA axis two; df = 1, *χ* ^2^ = 0.23, *P* = 0.63). Similarly, when Ericaceae specialists were excluded from the data, the relative proportion of cavity nesting bee species remained stable along the gradients in large wood boring beetle species richness (df = 1, *χ* ^2^ = 0.86, *P* = 0.35) and abundance (df = 1, *χ* ^2^ = 0.01, *P* = 0.93), among sampling years (df = 2, *χ* ^2^ = 0.49, *P* = 0.78), PCA axis one (df = 1, *χ* ^2^ = 2.80, *P* = 0.09) and two (df = 1, *χ* ^2^ < 0.01, *P* = 0.99).

**Figure 2:**
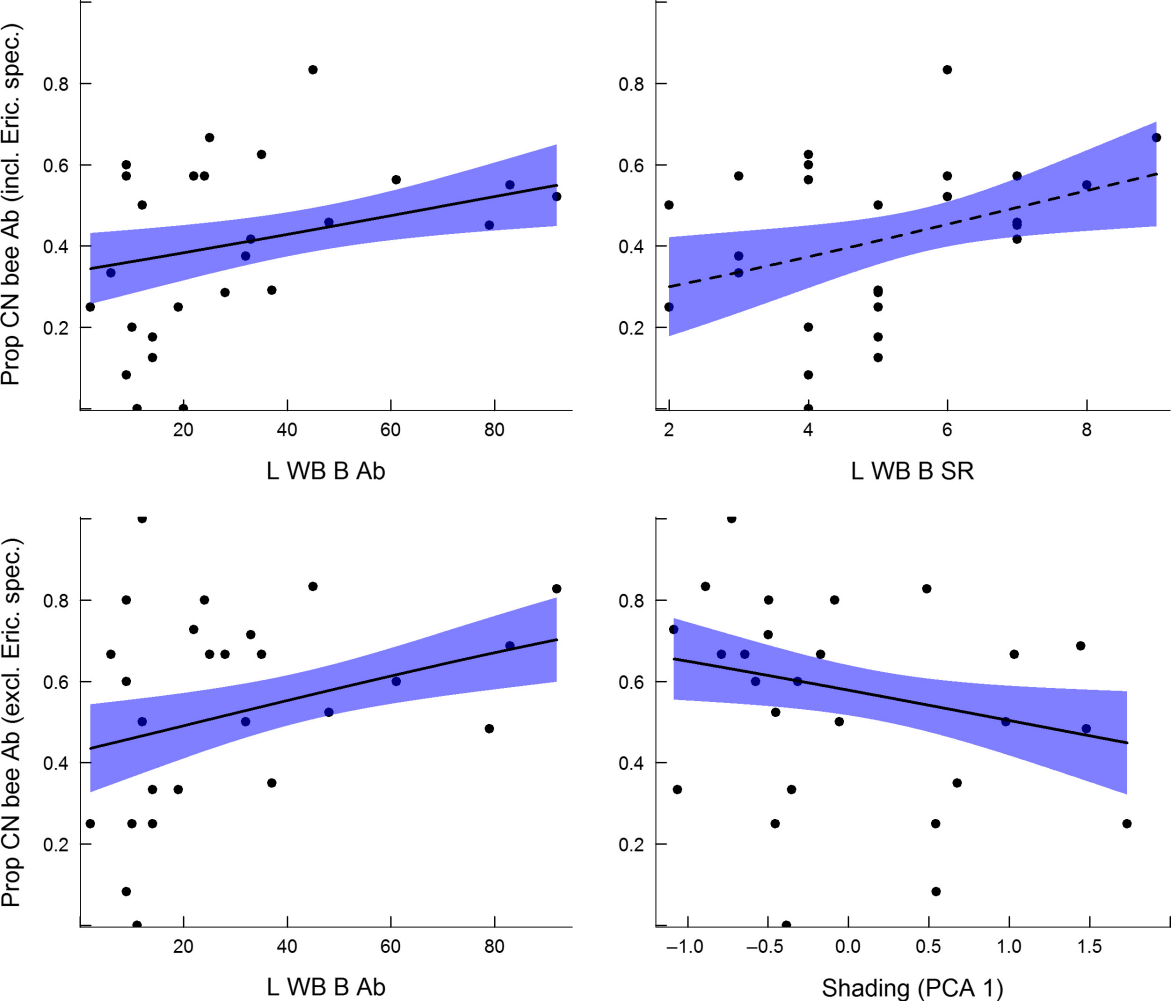
The proportion of cavity nesting (CN) bee species richness (SR) and abundance (Ab) increased with the Ab and SR of large wood boring beetles (L WB B) which excavate cavities with diameters ≥3* *mm. The proportion of cavity nesting bees in the bee species assemblage was calculated both with (incl. Eric. spec.) and without (excl. Eric. spec.) Ericaceae specialists. In both cases the L WB B Ab (fitted with solid lines) was the variable, that exerted the stongest influence on the bee response (see text and Table 2 for test statistics). However, the proportion of cavity nesting bee individuals also showed a decrease with the amount of shading (PCA one).

In contrast, the proportion of cavity nesting bee individuals (abundance) increased with the species richness (Fig. 2, df = 1, *χ* ^2^ = 5.08, *P* = 0.024) and abundance (Fig. 2, df = 1, *χ* ^2^ = 6.29, *P* = 0.012) of large wood boring beetles. The abundance of large wood boring beetles was the most important variable explaining the proportion of cavity nesting bee individuals (Table 2). There was no significant difference among sampling years (df = 2, *χ* ^2^ = 2.52, *P* = 0.28) or along PCA axis one (df = 1, *χ* ^2^ = 1.52, *P* = 0.22) and two (df = 1, *χ* ^2^ = 0.83, *P* = 0.36). The results were qualitatively similar when Ericaceae specialists were removed from the data, except from that the degree of tree regrowth and site productivity (PCA axes one and two) had a significant influence. The proportion of cavity nesting bee individuals increased with the species richness (Fig. 2, df = 1, *χ* ^2^ = 5.25, *P* = 0.022) and abundance (Fig. 2, df = 1, *χ* ^2^ = 7.94, *P* = 0.005) of large wood boring beetles and decreased with PCA axis one (Fig. 2, df = 1, *χ* ^2^ = 4.17, *P* = 0.041) but was not influenced by PCA axis two (df = 1, *χ* ^2^ = 0.11, *P* = 0.74) and did not differ among sampling years (df = 2, *χ* ^2^ = 1.67, *P* = 0.44). When included as explanatory variables in the same model the abundance of large wood boring beetles (df = 1, *χ* ^2^ = 13.07, *P* < 0.001) and PCA axis one (df = 1, *χ* ^2^ = 14.85, *P* < 0.001) remained statistically significant whereas the species richness of large wood boring beetles did not (df = 1, *χ* ^2^ = 0.23, *P* = 0.64). However, if the abundance of large wood boring beetles was not included in the model, both the species richness of large wood boring beetles (df = 1, *χ* ^2^ = 6.68, *P* = 0.010) and PCA axis one (df = 1, *χ* ^2^ = 5.60, *P* = 0.020) were significant. Thus, the most important predictors of change in the proportionate abundance of cavity nesting bees, when Ericaceae specialists were excluded, were the abundance of large wood boring beetles and PCA axis one (Fig. 2).

**Table 2:** The proportion of cavity nesting bee individuals in local bee species assemblages increased with the species richness and abundance of large wood boring beetles, which excavate cavities with diameters ≥3 mm. When Ericaceae specialists were excluded the proportion of cavity nesting bees also decreased with the degree of vegetation shading (PCA axis one) the ground. Models were fitted using binomial GLMs, Nagelkerke *R* ^2^ values are shown

	df	*β*	SE	*z* value	*R* ^2^	P‐value
Proportion of cavity nesting bee individuals (incl. Ericaceae specialists)						
Intercept	25	−1.179	0.437	−2.70		0.007
Large wood boring beetle species richness		0.166	0.074	2.23	0.22	0.026
Intercept	25	−0.662	0.204	−3.26		0.001
Large wood boring beetle abundance		0.009	0.004	2.49	0.27	0.013
Proportion of cavity nesting bee individuals (excl. Ericaceae specialists)						
Intercept	25	−0.773	0.472	−1.64		0.102
Large wood boring beetle species richness		0.184	0.081	2.27	0.21	0.023
Intercept	25	−0.285	0.232	−1.23		0.219
Large wood boring beetle abundance		0.012	0.005	2.77	0.31	0.006
Intercept	25	0.315	0.129	2.44		0.015
PCA axis one (shade)		−0.301	0.148	−2.03	0.17	0.042

### The functional diversity within beetle species assemblages drives the size diversity within bee species assemblages (Hypothesis 3)

**Figure 3:**
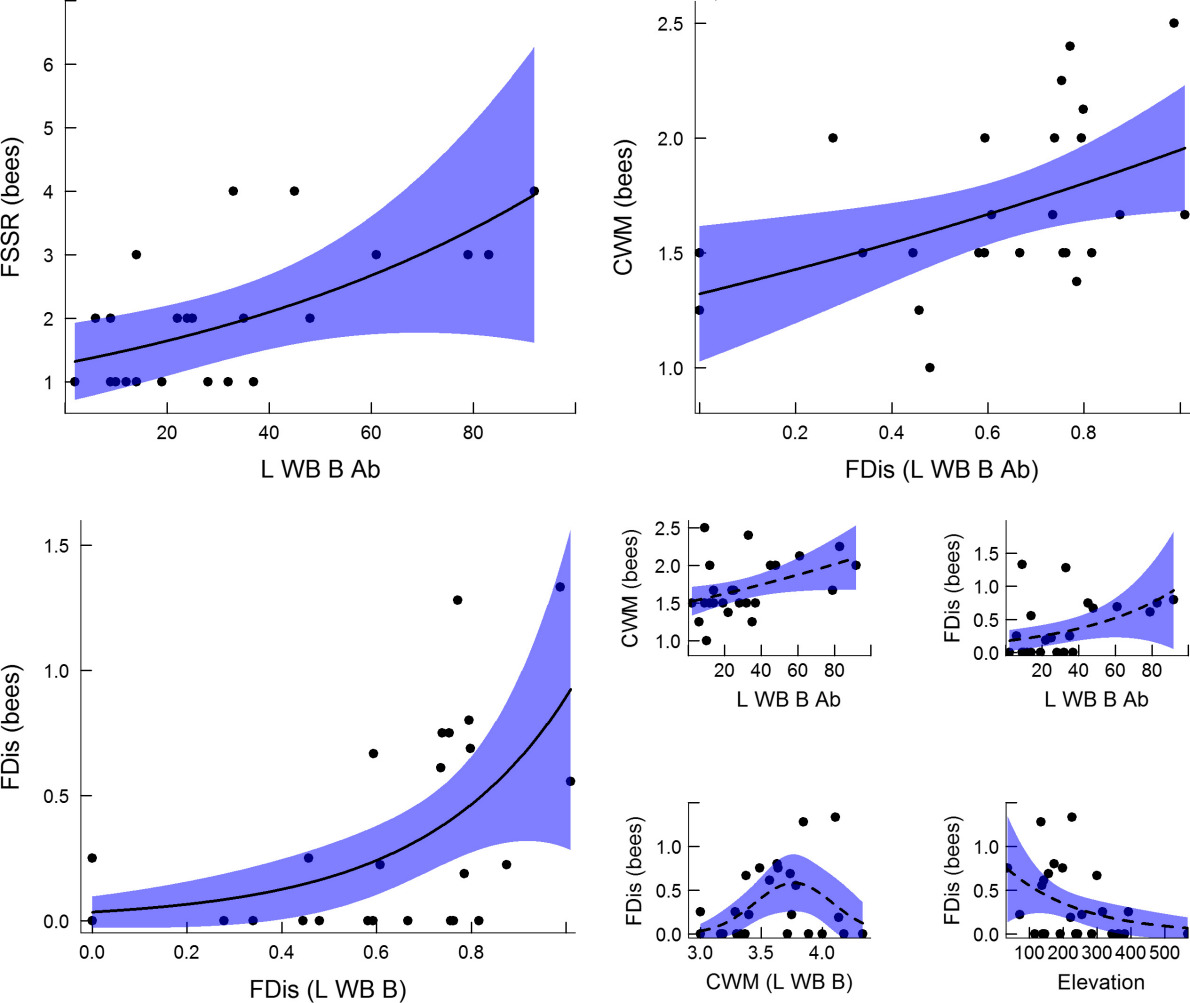
Enlarged plots showing the most important drivers of the functionally singular species richness (FSSR), community weighted mean (CWM) and functional dispersion (FDis) of the body size of cavity nesting bees. Relationships in reduced plots, with dashed regression lines, were significant when tested separately, but were not included following backward elimination of variables. The explanatory variables were the abundance, functional dispersion and community weighted mean of large wood boring beetles (L WB B) which excavate cavities with diameters ≥3 mm and elevation (m a.s.l.).

The size diversity of cavity‐nesting bees increased with species richness (SR), abundance (Ab) and functional diversity of wood boring beetles in the community (Table 3, Fig. 3). Specifically, the number of unique size clases (FSSR) of cavity‐nesting bees increased with the abundance of large wood boring beetles (df = 1, *χ* ^2^ = 5.40, *P* = 0.020). We found no significant influence of the large wood boring beetle (L WB B) species richness (df = 1, *χ* ^2^ = 3.37, *P* = 0.066), FDis (df = 1, *χ* ^2^ = 2.68, *P* = 0.101) and elevation (df = 1, *χ* ^2^ = 2.73, *P* = 0.098), although the p‐values were low. The FSSR_bees_ was not influenced by non‐wood boring beetles (SR; df = 1, *χ* ^2^ = 0.09, *P* = 0.759, Ab; df = 1, *χ* ^2^ = 0.56, *P* = 0.456), small wood boring beetles (SR; df = 1, *χ* ^2^ = 0.07, *P* = 0.798, Ab; df = 1, *χ* ^2^ = 0.01, *P* = 0.927), FSSR_L WB B_ (df = 1, *χ* ^2^ = 0.33, *P* = 0.566), CWM_L WB B_ (df = 2, *χ* ^2^ = 3.90, *P* = 0.142) or sampling year (df = 2, *χ* ^2^ = 2.53, *P* = 0.282). The strongest relationship was found between the functionally singular species richness of cavity nesting bees and the abundance of large wood boring beetles, and the latter was the only explanatory variable included in the final model following backward elimination (Fig. 3, Table 3).

**Table 3:** Final models for the size diversity of cavity nesting bees following backward elimination of the explanatory variables with a *P*‐value <0.10 (Table S6, Supporting information). Models were fitted using Poisson (Functionally singular species richness_bees_) and Quasipoisson (Community weighted mean_bees_ and Functional dispersion_bees_) generalized linear models (GLMs). Large wood boring beetle species excavate cavities with diameters ≥3 mm which may be used as nest sites by cavity nesting bees. Nagelkerke *R* ^2^ values are shown

	df	*β*	SE	*z* value	*R* ^2^	*P*‐value
Functionally singular species richness_bees_						
Intercept	23	0.253	0.243	1.04		0.298
Large wood boring beetle abundance		0.012	0.005	2.42	0.48	0.015
Community weighted mean_bees_						
Intercept	23	0.279	0.114	2.44		0.023
Functional dispersion_beetles_		0.389	0.164	2.36	0.21	0.027
Functional dispersion_bees_						
Intercept	23	−3.391	0.948	−3.58		0.002
Functional dispersion_beetles_		3.278	1.199	2.73	0.33	0.012

The community‐weighted mean body‐size of bees (CWM_bees_) increased with the abundance (Fig. 3, df = 1, scaled deviance (D) = 4.91, *P* = 0.027) and FDis (Fig. 3, df = 1, D = 5.78, *P* = 0.016) of large wood boring beetles. Although not statistically significant the results for the influence of the CWM_L WB B_ (df = 2, D = 4.59, *P* = 0.101) and elevation (df = 1, D = 2.71, *P* = 0.100) also suggested a trend. The CWM_bees_ was not influenced by the species richness (df = 1, D = 1.38, *P* = 0.239) or the FSSR_L WB B_ (df = 1, D = 0.03, *P* = 0.872) of large wood boring beetles. Nor was there any influence of non‐wood boring beetles (SR; df = 1, D = 1.38, *P* = 0.240, Ab; df = 1, D = 0.10, *P* = 0.750), small wood boring beetles (SR; df = 1, D = 1.20, *P* = 0.273, Ab; df = 1, D = 0.358, *P* = 0.550) or among year differences (df = 2, D = 3.63, *P* = 0.163). The FDis_L WB B_ was the strongest explanatory variable and the only explanatory variable retained in the model following backward elimination of variables (Fig. 3, Table 3).

The functional dispersion in terms of bee body‐sizes (FDis_bees_) decreased with elevation (Fig. 3, df = 1, D = 4.08, *P* = 0.044), increased with the abundance of large wood boring beetles (Fig. 3, df = 1, D = 4.14, *P* = 0.042), showed a hump‐backed relationship with the community‐weighted mean diameter‐class (Fig. 3, df = 2, D = 6.41, *P* = 0.041) of large wood boring beetles and increased with the functional dispersion of wood boring beetles (Fig. 3, df = 1, D = 9.58, *P* = 0.002). The FDis_bees_ was unaffected by the species richness (df = 1, D = 2.30, *P* = 0.129) and FSSR (df = 1, D = 0.07, *P* = 0.789) of large wood boring beetles. Also, the FDis_bees_ was unaffected by the species richness and abundance of non‐wood boring beetles (SR; df = 1, D = 0.10, *P* = 0.758, Ab; df = 1, D = 0.16, *P* = 0.69) and small wood boring beetles (SR; df = 1, D = 0.23, *P* = 0.63, Ab; df = 1, D = 0.13, *P* = 0.72). There was no significant change in the FDis between years (df = 2, D = 2.74, *P* = 0.25). The FDis_L WB B_ was the only variable included in the final model after backward elimination of variables (Fig. 3, Table 3).

The different measures of the size diversity within cavity‐nesting bee species assemblages were highly correlated, showing that the most diverse bee communities, in terms of body sizes, had more large bees (namely Megachilids), while still containing the smaller *Hylaeus* species. Specifically, FSSR_bees_ increased with both the FDis_bees_ (Spearman's rank correlation; rho = 0.93, *P* < 0.001) and CWM_bees_ (rho = 0.61, *P* = 0.001). Similarly, the FDis_bees_ also increased with the CWM_bees_ (rho = 0.70, *P* < 0.001). Moreover, the species richness of cavity nesting bees was also strongly related to FSSR_bees_ (rho = 0.90, *P* < 0.001), FDis_bees_ (rho = 0.84, *P* < 0.001) and CWM_bees_ (rho = 0.50, *P* < 0.011).

## Discussion

Previous studies have identified the importance of nesting substrates (Potts et al. 2005; Murray et al. 2012) in organizing bee species assemblages. Moreover, it has recently been shown that cavities produced by wood boring beetles provide nest sites for secondary nesting Aculeates, including cavity nesting bees, but that the occupation of nest sites depends on substrate characteristics (Westerfelt et al. 2015). The present study shows that the species diversity of cavity‐nesting bees is related to the species diversity of cavity‐producing beetles and that this relationship can be explained by the size diversity within both taxa (Tables 2, 3, Figs 2, 3).

Importantly, the abundance of large wood boring beetles was a more important determinant of cavity nesting bee species richness and abundance than the abiotic filter elevation (Fig. 1, Table S5, Supporting information). This is somewhat surprising, because elevation has been shown to exert a strong influence on bee species assemblages (Hoiss et al. 2012; Sydenham et al. 2015). However, the elevational gradient in the present study may not have been long enough to enforce the strong filtering effect found by Hoiss et al. (2012). Yet the elevational gradient did drive a synchronous decrease in both cavity nesting bee species richness (Fig. 1, Table S5, Supporting information) and large wood boring beetles (species richness vs. elevation; rho = −0.43, *P* = 0.001, abundance vs. elevation; rho = −0.40, *P* = 0.003) suggesting that elevation did pose a filtering effect on the entire, beetle and bee, community. Some of the variation in bee species richness explained by large wood boring beetles may therefore have been driven by a synchronous decline with elevation. Even so, the variance explained by large wood boring beetles was larger than that of elevation suggesting a substantial influence of wood boring beetles per se.

Furthermore, that the cavity nesting bee species richness and abundance were not significantly related to the species richness and abundance of small and non‐wood boring beetles and that cavity nesting bees and large wood boring beetles did not co‐vary with habitat area also suggests that there was a guild specific link between cavity nesting bees and large wood boring beetles. The hypothesis that the relationship between cavity nesting bees and large wood boring beetles was driven by nest site facilitation rather than by a shared response to the availability of forage resources was supported by an increase in the proportion of cavity nesting bee individuals with the abundance and species richness of large wood boring beetles (Table 2). Indeed, the estimated proportion of cavity nesting bee individuals increased with the abundance of large wood boring beetles (min = 2, max = 92) from 34% to 55% when Ericaceae specialists were included and from 44% to 70% when specialists were excluded (Table 2, Fig. 2). Importantly, this relationship was not an artefact of the productivity and regrowth in the site as the positive relationship between large wood boring beetles and the proportion of cavity nesting bees remained significant when shade (PCA axis one) was included as explanatory variable in the model. That shade was related to a decrease in the proportion of cavity nesting bees may have been due to an increased abundance of ground nesting bees in areas with a high site index. This would be expected if soils deep enough for ground nesting bees to nest in are mainly found in the more productive sites in our region. That secondary cavity‐nesters depend on cavity‐excavators has previously been shown for other functional groups of bees such as ground nesting bumblebees (*Bombus* sp.) that nest in rodent holes (McFrederick and LeBuhn 2006a,b) as well as for the variety of non‐bee taxa dependent on abandoned wood‐pecker nests (Martin and Eadie 1999). Although our findings are based on correlative and not experimental evidence, our findings concur with the results of an experiment showing that the availability of nesting resources pose a major limiting factor in the common cavity‐nesting bee *Osmia bicornis* (Syn. = *O. rufa*) (Steffan‐Dewenter and Schiele 2008) and also that nesting substrates are important drivers of bee diversity (Potts et al. 2005; Murray et al. 2012). Moreover, of the cavity‐nesting bee species found in this study only *Megachile nigriventris* is able to excavate their own cavities in dead wood (Westrich 1989). It is therefore unlikely that the observed increase of cavity‐nesting bees with large wood boring beetles was caused by both species groups responding in similar ways to the availability of dead wood, an important driver of beetle diversity (Grove 2002; Lachat et al. 2012).

The relationships between the functional diversity of large wood boring beetles and cavity nesting bees suggest a mechanistic link between the two groups (Fig. 3, Table 3). Indeed, Westerfelt et al. (2015) found that the diameter of nest holes was an important determinant of nest occupancy by cavity nesting Aculeates. That the number of different size classes (FSSR) of cavity‐nesting bees increased with the abundance of large wood boring beetles suggests that the density of nest holes is an important driver of the functional composition of cavity nesting bee species assemblages. This was also supported by the finding that the community‐weighted mean bee size (CWM) increased with the functional dispersion (FDis) of large wood boring beetles, suggesting that the largest bees were only able to find suitable nesting sites in the most functionally diverse beetle assemblages. Indeed, the functional dispersion (FDis) of cavity nesting bees also increased with the most functionally diverse beetle species assemblage. Interestingly, the FDis_bees_ showed a humpbacked relationship to the CWM of large wood boring beetles (Fig. 3, Table S6, Supporting information). This pattern would be expected if intermediate values of CWM_L WB B_ indicated that all functional types of large wood boring beetles were present and equally common within an area, thereby supporting a high diversity of nesting opportunities for cavity nesting bees. In contrast, if low or high values of CWM_L WB B_ indicates the dominance of either, relatively, small or large, large wood boring beetles, it might indicate situations where not all niches are supported for cavity nesting bees. This would be in line with Grime's (1998) “biomass ratio hypothesis” that the impact of species on ecosystem functioning is proportionate to their abundance, so that the most dominant species are the most influential.

## Conclusions

The role of facilitation in organizing communities has traditionally received less attention than competitive and trophic interactions (Bruno et al. 2003). This study documents a strong relationship between wood boring beetles and cavity nesting bees suggesting that non‐trophic facilitative interactions among species assemblages likely play a significant role in maintaining both size and species diversity within cavity nesting bee species assemblages. Indeed, identifying and managing for such interactions may be of high importance for restoration ecology (Byers et al. 2006) and conservation biology (Martin and Eadie 1999).

## Data Accessibility

Data available from the Dryad Digital Repository: http://dx.doi.org/10.5061/dryad.5r6pq

## Conflict of Interest

None declared.

## Supporting information


**Figure S1.** Location of study sites in south east Norway.

**Table S1**. Results from Principal Components Analysis (PCA) on variables related to regrowth and productivity (i.e. shading) within power line clearings.

**Table S2**. List of the species richness and abundance of large wood boring beetles and cavity nesting bees and the diameter of the exit holes they produce and widths of their thorax (ITD).

**Table S3**. Spearman's rank correlation among explanatory variables.

**Table S4**. Solitary bees sampled in power line clearings.

**Table S5**. Outputs from GLMs on the species richness and abundance of cavity nesting bees and all explanatory variables tested individually as well as the full models.

**Table S6**. Outputs from GLMs on the size diversity of cavity nesting bees and all explanatory variables tested individually as well as the full models.
