# Supplementary figures and images for: Inter‐assemblage facilitation: the functional diversity of cavity‐producing beetles drives the size diversity of cavity‐nesting bees
Source: Ecol Evol. 2016 Jan 8;6(2):412–25. doi: 10.1002/ece3.1871 (PMC4729264; doi:10.1002/ece3.1871)

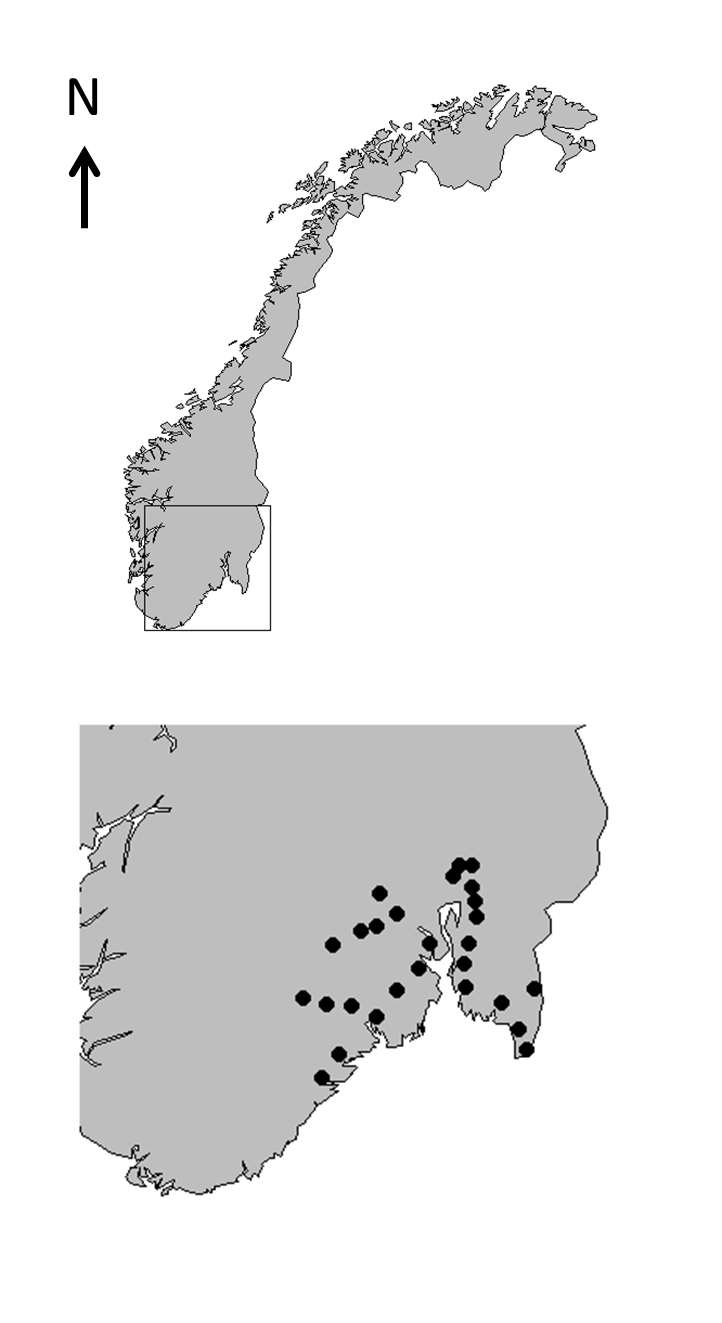

Supplement: Supplementary file 1 — Figure S1. Location of study sites in south east Norway. [file ECE3-6-412-s001.tif]
