# Supplementary material for: Inter‐assemblage facilitation: the functional diversity of cavity‐producing beetles drives the size diversity of cavity‐nesting bees
Source: Ecol Evol. 2016 Jan 8;6(2):412–25. doi: 10.1002/ece3.1871 (PMC4729264; doi:10.1002/ece3.1871)
Supplement: Supplementary file 2 — Table S1. Results from Principal Components Analysis (PCA) on variables related to regrowth and productivity (i.e. shading) within power line clearings. [file ECE3-6-412-s002.docx]

| PCA Gradient characteristics | | | |  |  |  |  |
| --- | --- | --- | --- | --- | --- | --- | --- |
|  |  | PC1 | PC2 | PC3 | PC4 | PC5 | PC6 |
|  | Eigenvalue | 5.54 | 2.47 | 1.10 | 0.90 | 0.46 | 0.24 |
|  | Proportion Explained | 0.50 | 0.23 | 0.10 | 0.08 | 0.04 | 0.02 |
|  | Cumulative Proportion | 0.50 | 0.73 | 0.83 | 0.91 | 0.95 | 0.97 |
|  |  |  |  |  |  |  |  |
| Loadings | |  |  |  |  |  |  |
|  |  | PC1 | PC2 | PC3 | PC4 | PC5 | PC6 |
|  | Relascope sum | 0.95 | -0.52 | 0.01 | 0.27 | 0.36 | -0.16 |
|  | No. trees | 1.08 | 0.43 | -0.19 | 0.18 | -0.32 | -0.02 |
|  | No. coniferous trees | 0.56 | -0.94 | -0.56 | -0.07 | -0.12 | 0.10 |
|  | No. deciduous trees | 0.96 | 0.68 | -0.05 | 0.20 | -0.30 | -0.04 |
|  | No. Spruce trees | 0.54 | 0.16 | -0.72 | -0.83 | 0.05 | -0.09 |
|  | No. Pine trees | 0.31 | -1.10 | -0.22 | 0.36 | -0.16 | 0.16 |
|  | Mean tree height | 1.09 | -0.18 | 0.45 | -0.19 | -0.08 | 0.15 |
|  | Mean tree crown width | 0.87 | -0.24 | 0.64 | -0.46 | -0.22 | 0.11 |
|  | Max tree height | 1.05 | -0.29 | 0.30 | -0.14 | 0.38 | -0.16 |
|  | Sum crown width | 1.08 | 0.28 | -0.16 | 0.33 | -0.04 | -0.24 |
|  | Site index | 0.77 | 0.73 | -0.23 | 0.16 | 0.39 | 0.43 |

Table S1 Results from Principal components analysis (PCA) on the degree of regrowth and productivity (site index) within power line clearings. See Methods and Eldegard et al. (2015) for details.

References

Eldegard, K., Totland, Ø. & Moe, S. R. (2015). Edge effects on plant communities along power line clearings. *Journal of Applied Ecology* (in press DOI: 10.1111/1365-2664.12460).
