# Supplementary material for: Inter‐assemblage facilitation: the functional diversity of cavity‐producing beetles drives the size diversity of cavity‐nesting bees
Source: Ecol Evol. 2016 Jan 8;6(2):412–25. doi: 10.1002/ece3.1871 (PMC4729264; doi:10.1002/ece3.1871)
Supplement: Supplementary file 3 — Table S2. List of the species richness and abundance of large wood boring beetles and cavity nesting bees and the diameter of the exit holes they produce and widths of their thorax (ITD). [file ECE3-6-412-s003.docx]

Table S2. The number of individuals and diameter class of large wood boring beetles and the number of individuals of cavity-nesting bees. The number of individuals and diameter class of large wood boring beetles and the number of individuals of cavity-nesting bees. The diameter class shows the diameter, in millimetres, of exit holes produced by adult beetles when leaving their nest chamber and the width of the thorax, inter-tegulae distance (ITD), of the cavity nesting bees in millimetres. ITD n shows the number of individuals used to calculate the average ITD and its standard deviation (ITD SD.)

| Order | Family | Species | Individuals | Diameter Class | ITD n | ITD SD. |
| --- | --- | --- | --- | --- | --- | --- |
| Coleoptera | Anthribidae | *Platystomus albinus* | 3 | 4 |  |  |
|  | Buprestidae | *Anthaxia quadripunctata* | 38 | 3 |  |  |
|  |  | *Buprestis octoguttata* | 19 | 5 |  |  |
|  | Cerambycidae | *Aegomorphus clavipes* | 2 | 6 |  |  |
|  |  | *Alosterna tabacicolor* | 1 | 3 |  |  |
|  |  | *Anoplodera maculicornis* | 101 | 4 |  |  |
|  |  | *Anoplodera rubra* | 100 | 5 |  |  |
|  |  | *Anoplodera sanguinolenta* | 81 | 3 |  |  |
|  |  | *Arhopalus rusticus* | 1 | 5 |  |  |
|  |  | *Clytus arietis* | 15 | 3 |  |  |
|  |  | *Leptura maculata* | 1 | 5 |  |  |
|  |  | *Leptura melanura* | 351 | 3 |  |  |
|  |  | *Leptura nigra* | 1 | 3 |  |  |
|  |  | *Leptura quadrifasciata* | 70 | 5 |  |  |
|  |  | *Pogonocherus fasciculatus* | 2 | 3 |  |  |
|  |  | *Saperda scalaris* | 1 | 5 |  |  |
|  |  | *Tetropium castaneum* | 2 | 4 |  |  |
|  | Curculionidae | *Pissodes pini* | 2 | 5 |  |  |
| Hymenoptera |  |  |  |  |  |  |
|  | Colletidae | *Hylaeus angustatus* | 7 | 0.9 = 1* | 5 | 0.04 |
|  |  | *Hylaeus annulatus* | 17 | 1.5 = 1.5* | 10 | 0.10 |
|  |  | *Hylaeus brevicornis* | 2 | 0.95 = 1* | 10 | 0.04 |
|  |  | *Hylaeus communis* | 24 | 1.4 = 1.5* | 10 | 0.05 |
|  |  | *Hylaeus confusus* | 69 | 1.5 = 1.5* | 10 | 0.10 |
|  |  | *Hylaeus hyalinatus* | 7 | 1.2 = 1* | 10 | 0.13 |
|  |  | *Hylaeus rinki* | 1 | 1.15 = 1* | 2 | 0.05 |
|  | Megachilidae | *Hoplitis tuberculata* | 1 | 2.2 = 2* | 10 | 0.17 |
|  |  | *Megachile nigriventris* | 2 | 4.3 = 4.5* | 3 | 0.13 |
|  |  | *Megachile versicolor* | 1 | 3.3 = 3.5* | 10 | 0.16 |
|  |  | *Osmia bicornis* | 1 | 3.1 = 3* | 10 | 0.13 |
|  |  | *Osmia caerulescens* | 1 | 2.5 = 2.5* | 8 | 0.22 |
|  |  | *Osmia nigriventris* | 8 | 3.3 = 3.5* | 8 | 0.16 |
|  |  | *Osmia parietina* | 4 | 2.1 = 2* | 9 | 0.16 |
|  |  | *Osmia uncinata* | 2 | 2.5 = 2.5* | 3 | 0.11 |
| * Measurements of thorax width were rounded to the nearest 0.5 mm to account for intra-specific variation and potential measurement errors (ITD SD). The rounded values were used in the analyses of functional bee diversity. | | | | | | |
