# Supplementary material for: Inter‐assemblage facilitation: the functional diversity of cavity‐producing beetles drives the size diversity of cavity‐nesting bees
Source: Ecol Evol. 2016 Jan 8;6(2):412–25. doi: 10.1002/ece3.1871 (PMC4729264; doi:10.1002/ece3.1871)
Supplement: Supplementary file 4 — Table S3. Spearman's rank correlation among explanatory variables. [file ECE3-6-412-s004.docx]

Table S3. Spearman’s rank correlations (rho) among explanatory variables from 27 power line clearings. The non-abbreviated form of the variables; species richness (SR), abundance (Ab) large wood boring beetle (L WB B), functionally singular species richness (FSSR), functional dispersion (FDis), community weighted mean (CWM), non-wood boring beetles (N WB B) and small wood boring beetles (S WB B). Large wood boring beetles excavate cavities with diameters larger than 3 mm, which may be occupied by cavity nesting bees.

|  | L WB B Ab | | L WB B SR | | L WB B FGR | | L WB B FDis | | L WB B CWM | |
| --- | --- | --- | --- | --- | --- | --- | --- | --- | --- | --- |
|  | rho | p | rho | p | rho | p | rho | p | rho | p |
| L WB B SR | 0.65 | <0.01 |  |  |  |  |  |  |  |  |
| L WB B FSSR | 0.49 | 0.01 | 0.69 | <0.01 |  |  |  |  |  |  |
| L WB B FDis | 0.33 | 0.09 | 0.41 | 0.03 | 0.46 | 0.02 |  |  |  |  |
| L WB B CWM | 0.18 | 0.36 | 0.39 | 0.04 | 0.50 | 0.01 | 0.78 | <0.01 |  |  |
| N WB B Ab | 0.28 | 0.16 | 0.35 | 0.08 | 0.16 | 0.41 | 0.14 | 0.49 | 0.09 | 0.67 |
| N WB B SR | 0.06 | 0.76 | 0.28 | 0.15 | 0.01 | 0.97 | 0.10 | 0.63 | -0.06 | 0.76 |
| S WB B Ab | 0.01 | 0.97 | 0.09 | 0.67 | 0.27 | 0.18 | -0.29 | 0.14 | -0.19 | 0.34 |
| S WB B SR | 0.13 | 0.53 | 0.47 | 0.01 | 0.62 | <0.01 | 0.03 | 0.89 | 0.16 | 0.42 |
| Elevation | -0.40 | 0.04 | -0.43 | 0.03 | -0.17 | 0.40 | -0.43 | 0.03 | -0.32 | 0.11 |
|  |  |  | N WB B Ab | | N WB B SR | | S WB B Ab | | S WB B SR | |
|  |  |  | rho | p | rho | p | rho | p | rho | p |
| N WB B SR | |  | 0.74 | <0.01 |  |  |  |  |  |  |
| S WB B Ab | |  | 0.21 | 0.29 | 0.22 | 0.28 |  |  |  |  |
| S WB B SR | |  | 0.22 | 0.27 | 0.14 | 0.48 | 0.53 | 0.00 |  |  |
| Elevation |  |  | -0.34 | 0.09 | -0.29 | 0.14 | 0.42 | 0.03 | -0.12 | 0.56 |
