# Supplementary material for: Inter‐assemblage facilitation: the functional diversity of cavity‐producing beetles drives the size diversity of cavity‐nesting bees
Source: Ecol Evol. 2016 Jan 8;6(2):412–25. doi: 10.1002/ece3.1871 (PMC4729264; doi:10.1002/ece3.1871)
Supplement: Supplementary file 5 — Table S4. Solitary bees sampled in power line clearings. [file ECE3-6-412-s005.docx]

Table S4. Solitary bees sampled in 27 sites in power line clearings (Fig. 1). The table shows the number of species and individuals for each genus and the number of individuals of the most abundant species within each genus.

| Family | Genus | Species | Individuals | Most abundant species | Individuals |
| --- | --- | --- | --- | --- | --- |
| Andrenidae | |  |  |  |  |
|  | *Andrena* | 13 | 124 | *A. lapponica* | 64 |
| Colletidae | |  |  |  |  |
|  | *Colletes* | 2 | 2 | *C. daviesanus* | 1 |
|  | *Hylaeus* | 7 | 124 | *H. confusus* | 69 |
| Halictidae | |  |  |  |  |
|  | *Halictus* | 1 | 4 | *H. rubicundus* | 4 |
|  | *Lasioglossum* | 6 | 54 | *L. leucopus* | 24 |
|  | *Sphecodes* | 3* | 5 | *S. hyalinatus* | 3 |
| Megachilidae | |  |  |  |  |
|  | *Anthidium* | 1 | 1 | *A. punctatum* | 1 |
|  | *Hoplitis* | 1 | 1 | *H. tuberculata* | 1 |
|  | *Megachile* | 2 | 3 | *M. nigriventris* | 2 |
|  | *Osmia* | 5 | 16 | *O. nigriventris* | 8 |
|  | *Stelis* | 1 | 1 | *S. ornatula* | 1 |
| Apidae |  |  |  |  |  |
|  | *Nomada* | 5 | 14 | *N. panzeri* | 7 |
| * One *Sphecodes* specimen was too damaged for identification and was assigned to a morpho species | | | | | |
