# Supplementary material for: Inter‐assemblage facilitation: the functional diversity of cavity‐producing beetles drives the size diversity of cavity‐nesting bees
Source: Ecol Evol. 2016 Jan 8;6(2):412–25. doi: 10.1002/ece3.1871 (PMC4729264; doi:10.1002/ece3.1871)
Supplement: Supplementary file 6 — Table S5. Outputs from GLMs on the species richness and abundance of cavity nesting bees and all explanatory variables tested individually as well as the full models. [file ECE3-6-412-s006.docx]

Table S5.1. Relationships between the species richness (SR) and abundance (Ab) of cavity nesting bees and the diversity of beetles, elevation and sampling year. Explanatory variables were the species richness and abundance of large wood boring beetles (L WB B), small wood boring beetles (S WB B) and non-wood boring beetles (N WB B) and the elevation of the study site and the year the sampling was conducted at each site. Large wood boring beetles excavate cavities with diameters larger than three mm. Analyses were performed using Poisson generalized linear models (cavity nesting bee SR) and negative binomial regressions (cavity nesting bee Ab). Nagelkerke *R*^2^-values are shown.

|  |  | DF | β | | SE | z value | *R*^2^ | p-value |
| --- | --- | --- | --- | --- | --- | --- | --- | --- |
| Cavity nesting bee SR | |  | |  |  |  |  |  |
|  | Intercept | 25 | -0.118 | | 0.392 | -0.30 |  | 0.763 |
|  | L WB B SR |  | 0.200 | | 0.067 | 3.01 | 0.42 | 0.003 |
|  |  |  |  | |  |  |  |  |
|  | Intercept | 25 | 0.417 | | 0.206 | 2.03 |  | 0.043 |
|  | L WB B Ab |  | 0.015 | | 0.004 | 3.65 | 0.54 | <0.001 |
|  |  |  |  | |  |  |  |  |
|  | Intercept | 25 | 0.470 | | 0.331 | 1.42 |  | 0.156 |
|  | S WB B SR |  | 0.053 | | 0.034 | 1.57 | 0.12 | 0.116 |
|  |  |  |  | |  |  |  |  |
|  | Intercept | 25 | 0.839 | | 0.168 | 4.99 |  | <0.001 |
|  | S WB B Ab |  | 0.002 | | 0.002 | 0.89 | 0.04 | 0.373 |
|  |  |  |  | |  |  |  |  |
|  | Intercept | 25 | 0.554 | | 0.548 | 1.01 |  | 0.312 |
|  | N WB B SR |  | 0.005 | | 0.006 | 0.73 | 0.03 | 0.468 |
|  |  |  |  | |  |  |  |  |
|  | Intercept | 25 | 0.504 | | 0.310 | 1.62 |  | 0.105 |
|  | N WB B Ab |  | 0.001 | | 0.001 | 1.58 | 0.13 | 0.114 |
|  |  |  |  | |  |  |  |  |
|  | Intercept | 25 | 1.506 | | 0.271 | 5.55 |  | <0.001 |
|  | Elevation |  | -0.003 | | 0.001 | -2.18 | 0.26 | 0.029 |
|  |  |  |  | |  |  |  |  |
|  | Intercept (2009) | 24 | 1.237 | | 0.180 | 6.89 |  | <0.001 |
|  | Year (2010) |  | -0.483 | | 0.302 | -1.60 |  | 0.11 |
|  | Year (2013) |  | -0.495 | | 0.283 | -1.75 | 0.21 | 0.08 |
|  |  |  |  | |  |  |  |  |
| Cavity nesting bee Ab | |  |  | |  |  |  |  |
|  | Intercept | 25 | -0.148 | | 0.520 | -0.29 |  | 0.776 |
|  | L WB B SR |  | 0.340 | | 0.094 | 3.62 | 0.46 | <0.001 |
|  |  |  |  | |  |  |  |  |
|  | Intercept | 25 | 0.658 | | 0.216 | 3.04 |  | 0.002 |
|  | L WB B Ab |  | 0.027 | | 0.005 | 5.73 | 0.80 | <0.001 |
|  |  |  |  | |  |  |  |  |
|  | Intercept | 25 | 1.144 | | 0.528 | 2.17 |  | 0.030 |
|  | S WB B SR |  | 0.063 | | 0.057 | 1.09 | 0.06 | 0.274 |
|  |  |  |  | |  |  |  |  |
|  | Intercept | 25 | 1.462 | | 0.264 | 5.54 |  | <0.001 |
|  | S WB B Ab |  | 0.005 | | 0.004 | 1.19 | 0.06 | 0.233 |
|  |  |  |  | |  |  |  |  |
|  | Intercept | 25 | 1.364 | | 0.856 | 1.60 |  | 0.111 |
|  | N WB B SR |  | 0.004 | | 0.010 | 0.39 | 0.01 | 0.694 |
|  |  |  |  | |  |  |  |  |
|  | Intercept | 25 | 0.729 | | 0.462 | 1.58 |  | 0.114 |
|  | N WB B Ab |  | 0.002 | | 0.001 | 2.18 | 0.21 | 0.029 |
|  |  |  |  | |  |  |  |  |
|  | Intercept | 25 | 2.355 | | 0.421 | 5.60 |  | <0.001 |
|  | Elevation |  | -0.003 | | 0.002 | -1.80 | 0.16 | 0.071 |
|  |  |  |  | |  |  |  |  |
|  | Intercept (2009) | 24 | 2.147 | | 0.289 | 7.43 |  | <0.001 |
|  | Year (2010) |  | -0.825 | | 0.443 | -1.86 |  | 0.062 |
|  | Year (2013) |  | -0.760 | | 0.414 | -1.84 | 0.23 | 0.067 |
|  |  |  |  | |  |  |  |  |

Table S5.2 Full models on the species richness (SR) and abundance (Ab) of cavity nesting bees. Explanatory variables from the single variable models listed Table S6.1 with p-values from likelihood ratio tests ≤ 0.1 were included in the full models. Variables marked in bold were included in the final models following backward elimination of variables.

|  |  | DF | β | SE | z value | *R*^2^ | p-value |
| --- | --- | --- | --- | --- | --- | --- | --- |
| Cavity nesting bee SR | | 23 |  |  |  | 0.62 |  |
|  | Intercept |  | 0.144 | 0.598 | 0.24 |  | 0.810 |
|  | **L WB B AB** |  | **0.011** | **0.005** | **1.95** |  | **0.051** |
|  | L WB B SR |  | 0.106 | 0.083 | 1.28 |  | 0.201 |
|  | Elevation |  | -0.001 | 0.001 | -0.44 |  | 0.660 |
| Cavity nesting bee Ab | | 20 |  |  |  | 0.90 |  |
|  | Intercept |  | -0.734 | 0.665 | -1.10 |  | 0.270 |
|  | **L WB B AB** |  | **0.029** | **0.007** | **4.34** |  | **<0.001** |
|  | L WB B SR |  | 0.160 | 0.092 | 1.73 |  | 0.084 |
|  | N WB B Ab |  | <0.001 | 0.001 | 0.22 |  | 0.829 |
|  | Elevation |  | 0.001 | 0.001 | 0.43 |  | 0.664 |
|  | Year (2010) |  | 0.220 | 0.357 | 0.62 |  | 0.538 |
|  | Year (2013) |  | 0.600 | 0.458 | 1.31 |  | 0.190 |
