# Supplementary material for: Inter‐assemblage facilitation: the functional diversity of cavity‐producing beetles drives the size diversity of cavity‐nesting bees
Source: Ecol Evol. 2016 Jan 8;6(2):412–25. doi: 10.1002/ece3.1871 (PMC4729264; doi:10.1002/ece3.1871)
Supplement: Supplementary file 7 — Table S6. Outputs from GLMs on the size diversity of cavity nesting bees and all explanatory variables tested individually as well as the full models. [file ECE3-6-412-s007.docx]

Table S6.1. Relationships between the size diversity of cavity nesting bees and the nomenclatural and functional diversity of wood boring beetles. Explanatory variables were the functionally singular species richness (FSSR), Community-weighted mean (CWM) diameter class and the functional dispersion of diameter classes of large wood boring beetles (L WB B) and the species richness (SR) and abundance (Ab) of small (S WB B) and non-wood boring (N WB B) beetles. Large wood boring beetles excavate cavities which may be occupied by cavity nesting bees. Variables were tested individually using Poisson generalized linear models (GLMs) for bee FSSR and Quasipoisson GLMs for bee CWM and FDis. Nagelkerke *R*^2^-values are shown.

| Response | Explanatory variable | | | | | *Df.* | β | SE | z | R^2^ | P |
| --- | --- | --- | --- | --- | --- | --- | --- | --- | --- | --- | --- |
| Bee Functionally singular species richness | | | | | | | | | |  |  |
|  |  | |  | | | |  |  |  |  |  |
|  |  | | | | Intercept | 23 | 0.335 | 0.621 | 0.54 |  | 0.590 |
|  |  | | | | L WB B FSSR |  | 0.125 | 0.221 | 0.57 | 0.03 | 0.572 |
|  |  |  | | | | |  |  |  |  |  |
|  |  | | | Intercept | | 23 | 0.020 | 0.457 | 0.04 |  | 0.965 |
|  |  | | | L WB B FDis | |  | 0.997 | 0.636 | 1.57 | 0.25 | 0.117 |
|  |  |  | | | | |  |  |  |  |  |
|  |  | | | | Intercept | 22 | 0.628 | 0.150 | 4.18 |  | <0.001 |
|  |  | | | | L WB B CWM_β1_ |  | 0.282 | 0.836 | 0.34 |  | 0.736 |
|  |  | | | | L WB B CWM_β2_ |  | -1.571 | 0.863 | -1.82 | 0.35 | 0.069 |
|  |  | |  | | | |  |  |  |  |  |
|  |  | | | | Intercept | 23 | -0.095 | 0.457 | -0.21 |  | 0.838 |
|  |  | | | | L WB B SR |  | 0.145 | 0.079 | 1.85 | 0.31 | 0.065 |
|  |  | |  | | | |  |  |  |  |  |
|  |  | | | | Intercept | 23 | 0.253 | 0.243 | 1.04 |  | 0.297 |
|  |  | | | | L WB B Ab |  | 0.012 | 0.005 | 2.42 | 0.48 | 0.015 |
|  |  |  | | | | |  |  |  |  |  |
|  |  | | | | Intercept | 23 | 0.573 | 0.415 | 1.38 |  | 0.167 |
|  |  | | | | S WB B SR |  | 0.011 | 0.044 | 0.26 | 0.01 | 0.797 |
|  |  |  | | | | |  |  |  |  |  |
|  |  | | | | Intercept | 23 | 0.687 | 0.206 | 3.34 |  | 0.001 |
|  |  | | | | S WB B Ab |  | -0.0003 | 0.003 | -0.09 | <0.01 | 0.928 |
|  |  |  | | | | |  |  |  |  |  |
|  |  | | | | Intercept | 23 | 0.483 | 0.637 | 0.76 |  | 0.448 |
|  |  | | | | N WB B SR |  | 0.002 | 0.007 | 0.31 | 0.01 | 0.758 |
|  |  |  | | | | |  |  |  |  |  |
|  |  | | | | Intercept | 23 | 0.417 | 0.375 | 1.11 |  | 0.266 |
|  |  | | | | N WB B Ab |  | 0.001 | 0.001 | 0.76 | 0.05 | 0.450 |
|  |  |  | | | | |  |  |  |  |  |
|  |  | | | | Intercept (2009) | 22 | 0.965 | 0.218 | 4.42 |  | <0.001 |
|  |  | | | | Year (2010) |  | -0.480 | 0.353 | -1.36 |  | 0.174 |
|  |  | | | | Year (2013) |  | -0.454 | 0.338 | -1.34 | 0.24 | 0.179 |
|  |  |  | | | | |  |  |  |  |  |
|  |  | | | | Intercept | 23 | 1.147 | 0.314 | 3.66 |  | <0.001 |
|  |  | | | | Elevation |  | -0.002 | 0.001 | -1.60 | 0.25 | 0.110 |
| Bee CWM ITD | | | | | | | | | |  |  |
|  |  | | | | Intercept | 23 | 0.497 | 0.185 | 2.69 |  | 0.013 |
|  |  | | | | L WB B FSSR |  | 0.011 | 0.067 | 0.16 | <0.01 | 0.873 |
|  |  |  | | | | |  |  |  |  |  |
|  |  | | | Intercept | | 23 | 0.279 | 0.114 | 2.44 |  | 0.023 |
|  |  | | | L WB B FDis | |  | 0.389 | 0.164 | 2.36 | 0.21 | 0.027 |
|  |  |  | | | | |  |  |  |  |  |
|  |  | | | | Intercept | 22 | 0.522 | 0.042 | 12.46 |  | <0.001 |
|  |  | | | | L WB B CWM_β1_ |  | 0.266 | 0.217 | 1.23 |  | 0.231 |
|  |  | | | | L WB B CWM_β2_ |  | -0.381 | 0.217 | -1.76 | 0.18 | 0.093 |
|  |  | |  | | | |  |  |  |  |  |
|  |  | | | | Intercept | 23 | 0.379 | 0.134 | 2.83 |  | 0.009 |
|  |  | | | | L WB B SR |  | 0.029 | 0.025 | 1.15 | 0.06 | 0.251 |
|  |  | |  | | | |  |  |  |  |  |
|  |  | | | | Intercept | 23 | 0.413 | 0.067 | 6.21 |  | <0.001 |
|  |  | | | | L WB B Ab |  | 0.004 | 0.002 | 2.25 | 0.19 | 0.034 |
|  |  |  | | | | |  |  |  |  |  |
|  |  | | | | Intercept | 23 | 0.659 | 0.129 | 5.17 |  | <0.001 |
|  |  | | | | S WB B SR |  | -0.015 | 0.014 | -1.09 | 0.05 | 0.289 |
|  |  |  | | | | |  |  |  |  |  |
|  |  | | | | Intercept | 23 | 0.554 | 0.064 | 8.63 |  | <0.001 |
|  |  | | | | S WB B Ab |  | -0.001 | 0.001 | -0.59 | 0.02 | 0.561 |
|  |  |  | | | | |  |  |  |  |  |
|  |  | | | | Intercept | 23 | 0.747 | 0.192 | 3.89 |  | 0.001 |
|  |  | | | | N WB B SR |  | -0.003 | 0.002 | -1.17 | 0.06 | 0.254 |
|  |  |  | | | | |  |  |  |  |  |
|  |  | | | | Intercept | 23 | 0.561 | 0.117 | 4.79 |  | <0.001 |
|  |  | | | | N WB B Ab |  | -0.0001 | 0.0002 | -0.32 | 0.01 | 0.753 |
|  |  |  | | | | |  |  |  |  |  |
|  |  | | | | Intercept (2009) | 22 | 0.636 | 0.072 | 8.88 |  | <0.001 |
|  |  | | | | Year (2010) |  | -0.138 | 0.105 | -1.31 |  | 0.204 |
|  |  | | | | Year (2013) |  | -0.191 | 0.103 | -1.85 | 0.15 | 0.077 |
|  |  |  | | | | |  |  |  |  |  |
|  |  | | | | Intercept | 23 | 0.666 | 0.094 | 7.07 |  | <0.001 |
|  |  | | | | Elevation |  | -0.001 | 0.0004 | -1.63 | 0.11 | 0.117 |
| Bee FDis ITD | | | | | | | | | |  |  |
|  |  | |  | | | |  |  |  |  |  |
|  |  | | | | Intercept | 23 | -1.343 | 1.064 | -1.26 |  | 0.220 |
|  |  | | | | L WB B FSSR |  | 0.101 | 0.380 | 0.27 | <0.01 | 0.794 |
|  |  |  | | | | |  |  |  |  |  |
|  |  | | | Intercept | | 23 | -3.391 | 0.948 | -3.58 |  | 0.002 |
|  |  | | | L WB B FDis | |  | 3.278 | 1.199 | 2.73 | 0.33 | 0.012 |
|  |  |  | | | | |  |  |  |  |  |
|  |  | | | | Intercept | 22 | -1.308 | 0.298 | -4.39 |  | <0.001 |
|  |  | | | | L WB B CWM_β1_ |  | 2.233 | 1.654 | 1.35 |  | 0.191 |
|  |  | | | | L WB B CWM_β2_ |  | -3.385 | 1.725 | -1.96 | 0.27 | 0.062 |
|  |  | |  | | | |  |  |  |  |  |
|  |  | | | | Intercept | 23 | -2.221 | 0.844 | -2.63 |  | 0.015 |
|  |  | | | | L WB B SR |  | 0.214 | 0.140 | 1.52 | 0.12 | 0.142 |
|  |  | |  | | | |  |  |  |  |  |
|  |  | | | | Intercept | 23 | -1.746 | 0.458 | -3.82 |  | 0.001 |
|  |  | | | | L WB B Ab |  | 0.018 | 0.009 | 2.13 | 0.22 | 0.044 |
|  |  |  | | | | |  |  |  |  |  |
|  |  | | | | Intercept | 23 | -0.748 | 0.723 | -1.04 |  | 0.312 |
|  |  | | | | S WB B SR |  | -0.038 | 0.082 | -0.46 | 0.01 | 0.647 |
|  |  |  | | | | |  |  |  |  |  |
|  |  | | | | Intercept | 23 | -0.977 | 0.356 | -2.74 |  | 0.012 |
|  |  | | | | S WB B Ab |  | -0.002 | 0.006 | -0.35 | 0.01 | 0.732 |
|  |  |  | | | | |  |  |  |  |  |
|  |  | | | | Intercept | 23 | -0.748 | 1.075 | -0.70 |  | 0.494 |
|  |  | | | | N WB B SR |  | -0.004 | 0.013 | -0.31 | 0.01 | 0.762 |
|  |  |  | | | | |  |  |  |  |  |
|  |  | | | | Intercept | 23 | -1.312 | 0.658 | -1.99 |  | 0.058 |
|  |  | | | | N WB B Ab |  | 0.001 | 0.001 | 0.41 | 0.01 | 0.689 |
|  |  |  | | | | |  |  |  |  |  |
|  |  | | | | Intercept (2009) | 22 | -0.609 | 0.344 | -1.77 |  | 0.090 |
|  |  | | | | Year (2010) |  | -0.976 | 0.657 | -1.49 |  | 0.152 |
|  |  | | | | Year (2013) |  | -0.642 | 0.564 | -1.14 | 0.14 | 0.267 |
|  |  |  | | | | |  |  |  |  |  |
|  |  | | | | Intercept | 23 | -0.137 | 0.495 | -0.28 |  | 0.785 |
|  |  | | | | Elevation |  | -0.005 | 0.002 | -1.89 | 0.18 | 0.072 |

Table S6.2 Full models on Relationships between the size diversity of cavity nesting bees and the nomenclatural and functional diversity of wood boring beetles. Explanatory variables from the single variable models listed Table S7.1 with p-values from likelihood ratio tests ≤ 0.1 were included in the full models. Variables marked in bold were included in the final models following backward elimination of variables. Nagelkerke R^2^ for the full models are shown.

| Response | Explanatory variable | | *Df.* | β | SE | z | R^2^ | P |
| --- | --- | --- | --- | --- | --- | --- | --- | --- |
| Bee Functionally singular species richness | | | | | | |  |  |
|  |  | Intercept | 20 | -0.031 | 0.810 | -0.04 | 0.58 | 0.97 |
|  |  | L WB B SR |  | 0.041 | 0.103 | 0.40 |  | 0.688 |
|  |  | **L WB B Ab** |  | **0.008** | **0.006** | **1.32** |  | **0.187** |
|  |  | L WB B FDis |  | 0.499 | 0.771 | 0.65 |  | 0.518 |
|  |  | Elevation |  | -0.001 | 0.002 | -0.40 |  | 0.691 |
| Bee CWM ITD | | | *Df.* | β | SE | z | R^2^ | P |
|  |  | Intercept | 19 | 0.092 | 0.371 | 0.25 | 0.33 | 0.806 |
|  |  | L WB B Ab |  | 0.002 | 0.002 | 1.21 |  | 0.243 |
|  |  | **L WB B FDis** |  | **0.632** | **0.525** | **1.20** |  | **0.243** |
|  |  | L WB B CWM_β1_ |  | -0.398 | 0.562 | -0.71 |  | 0.488 |
|  |  | L WB B CWM_β2_ |  | 0.268 | 0.456 | 0.59 |  | 0.564 |
|  |  | Elevation |  | -0.0002 | 0.0004 | -0.46 |  | 0.653 |
| Bee FDis ITD | | | *Df.* | β | SE | z | R^2^ | P |
|  |  | Intercept | 19 | -5.858 | 2.553 | -2.30 | 0.51 | 0.033 |
|  |  | L WB B Ab |  | 0.016 | 0.011 | 1.55 |  | 0.139 |
|  |  | **L WB B FDis** |  | **6.583** | **3.396** | **1.94** |  | **0.068** |
|  |  | L WB B CWM_β1_ |  | -4.653 | 4.180 | -1.11 |  | 0.280 |
|  |  | L WB B CWM_β2_ |  | 3.100 | 3.576 | 0.87 |  | 0.397 |
|  |  | Elevation |  | -0.001 | 0.003 | -0.36 |  | 0.726 |
